# D-Allulose (D-Psicose) Biotransformation From Allitol by a Newly Found NAD(P)-Dependent Alcohol Dehydrogenase From *Gluconobacter frateurii* NBRC 3264 and the Enzyme Characterization

**DOI:** 10.3389/fmicb.2022.870168

**Published:** 2022-04-25

**Authors:** Xin Wen, Huibin Lin, Yuhang Ning, Guangwen Liu, Yilin Ren, Can Li, Chengjia Zhang, Jianqun Lin, Xin Song, Jianqiang Lin

**Affiliations:** ^1^State Key Laboratory of Microbial Technology, Shandong University, Qingdao, China; ^2^Shandong Academy of Chinese Medicine, Jinan, China; ^3^Qingdao Longding Biotech Limited Company, Qingdao, China; ^4^School of Biological Engineering, Qilu University of Technology, Jinan, China

**Keywords:** D-allulose, allitol, NAD(P)-dependent alcohol dehydrogenase, *Gluconobacter frateurii NBRC 3264*, biotransformation

## Abstract

The NAD(P)-dependent alcohol dehydrogenase (ADH) gene was cloned from *Gluconobacter frateurii* NBRC 3264 and expressed in *Escherichia coli* BL21 star (DE3). The expressed enzyme was purified and the characteristics were investigated. The results showed that this ADH can convert allitol into D-allulose (D-psicose), which is the first reported enzyme with this catalytic ability. The optimum temperature and pH of this enzyme were 50°C and pH 7.0, respectively, and the enzyme showed a maximal activity in the presence of Co^2+^. At 1 mM Co^2+^ and allitol concentrations of 50, 150, and 250 mM, the D-allulose yields of 97, 56, and 38%, respectively, were obtained after reaction for 4 h under optimal conditions, which were much higher than that obtained by using the epimerase method of about 30%.

## Introduction

D-Allulose (D-psicose), an epimer of D-fructose at the C3 position, is a kind of rare sugar according to the definition by the International Society of Rare Sugars (ISRS). D-Allulose is a low-energy sweet and is regarded as a potential substitute for sucrose as it has 70% of the relative sweetness but only 0.3% of the energy of sucrose (Zhang et al., 2015). More importantly, D-allulose has many important physiological functions, for example, blood glucose suppressive effect (EdyLiani et al., 2020), body fat accumulation inhibitive effect (Kim et al., 2017), reactive oxygen species scavenging effect (Li et al., 2018), and neuroprotective effect (Zhao et al., 2021). In addition, it has good properties for food industry applications, such as improving the gelling behavior and producing good flavor (Zhang et al., 2013). Importantly, it has been approved as “generally regarded as safe” (GRAS) by the Food and Drug Administration (FDA) of the United States, and has been allowed to be used as an ingredient in dietary supplements in the United States and some other countries.

**FIGURE 1:**
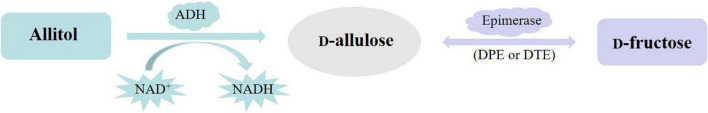
D-Allulose biotransformation from allitol or D-fructose (ADH, NAD(P)-dependent alcohol dehydrogenase; DPE, D-psicose 3-epimerase; DTE, D-tagatose 3-epimerase).

In nature, D-allulose is found in very small amounts in the wheat and *Itea* plants. So, it is impractical to extract it from natural resources for mass production of D-allulose. The chemical synthetic method is one choice, but it may produce toxic by-products and is not suitable for food production. Biotransformation is an ideal method and is widely accepted in D-allulose mass production due to the advantages of easy operation, mild reaction conditions, no toxic by-products, and environmental friendliness. At present, D-allulose was namely biotransformed from D-fructose by using D-psicose 3-epimerase or D-tagatose 3-epimerase (Zhu et al., 2012, 2019c; Li et al., 2018; Figure 1). However, the reaction catalyzed by epimerase is limited by thermodynamic equilibrium unfavorable to the D-allulose direction, and the conversion yield of D-allulose is about 30%, which greatly decreases the production efficiency and increases the difficulty in product separation. To overcome the limitation of thermodynamic equilibrium, Kim et al. added boronic acid to the reaction system to form a complex with sugar to increase the D-allulose conversion yield (Kim et al., 2008). As the binding affinity of boric acid to D-allulose is much higher than that of D-fructose, the reaction equilibrium is shifted toward the formation of D-allulose, and that increases the conversion yield of D-allulose (Kim et al., 2008). However, boric acid is toxic and used in large quantities, and the removal of boric acid is difficult. For the above reasons, this method is difficult to be applied in real applications. Alternatively, the thermodynamic equilibrium limitation can also be overcome by combining the D-allulose biocatalytic process with continuous D-allulose separation (Wagner et al., 2015; Li et al., 2021). However, this method is complex and cumbersome and is also difficult to be applied in real applications.

Fortunately, D-allulose can also be biotransformed from allitol by using dehydrogenation reaction using dehydrogenase as the catalyst according to the Izumoring strategy (Izumori, 2006), which can overcome the above limitation of the thermodynamic equilibrium and improve the conversion rate of D-allulose. Moreover, allitol can be prepared easily from low-cost substrates of D-glucose or D-fructose by the biotransformation method (Zhu et al., 2015; Hassanin et al., 2016; Wen et al., 2020a,b). Poonperm et al. (2007) biotransformed allitol into D-allulose by using the resting cells of *Bacillus pallidus* Y25 for the first time. Gullapalli et al. (2007). biotransformed allitol into D-allulose by using *Enterobacter aerogenes* IK7. However, the exact enzyme that catalyzed allitol into D-allulose was unknown.

In this study, the gene encoding NAD(P)-dependent alcohol dehydrogenase (ADH) with protein ID WP_099183078.1 from *Gluconobacter frateurii* NBRC 3264 was cloned and overexpressed in *E. coli*. The ADH was confirmed to convert allitol into D-allulose (D-psicose), which is the first reported enzyme with this catalytic ability. The enzymatic properties, such as optimal pH, temperature, and metal ion, of this ADH were investigated. The activation effect of Co^2+^ on the ADH to increase the enzyme activity and the D-allulose yield was determined, and the kinetics of this enzyme were also investigated. The highest D-allulose conversion yield of 97% was obtained, which was more than twofold higher than the epimerase method. The method developed in this study is expected to be applied to the industrial production of D-allulose.

## Materials and Methods

### Materials and Reagents

The restriction enzymes were obtained from TaKaRa (Beijing, China). The DNA polymerase was obtained from Vazyme (Nanjing, China). T_4_ DNA ligase was purchased from Thermo Fisher (United States). Ampicillin and isopropyl-β-D-1-thiogalactopyranoside (IPTG) were purchased from Sangon Biotech (Shanghai, China). Allitol was prepared in our lab as described previously (Wen et al., 2020a,b, 2022).

### Construction of Recombinant *E. coli* Expressing Alcohol Dehydrogenase

According to NCBI, the whole genome of *Gluconobacter frateurii* NBRC 3264 was sequenced by Hosoyama et al. and was released into the GenBank National Center for Biotechnology Information (NCBI)^1^. The *adh* gene locus_tag was GFR01_RS14945 and the ADH protein ID number was WP_099183078.1. The optimization and synthesis of the gene encoding NAD(P)-dependent alcohol dehydrogenase (ADH) were made by a company named Boshang (Jinan, China). The *adh* region was initially amplified from the plasmid pETDuet_–1_-*adh* (no 6 × His-tag) using primers *adh*-pET22b-*Nde* I-U and *adh*-pET22b-*Xho* I-D (Table 1). A 6 × His-tag sequence was present in the vector to aid protein purification. Then, the *adh* region was inserted into the plasmid pET22b at the *Nde* I and *Xho* I restriction sites to create the recombinant plasmid pET22b-*adh*. The recombinant plasmid pET22b-*adh* was transformed into *E. coli* DH5α and verified correctly by electrophoresis and sequencing. And then, the recombinant plasmid pET22b-*adh* was transformed into *E. coli* BL21 star (DE3) for the expression of ADH. The strains, plasmids, and primers used in this study are listed in Table 1.

**TABLE 1:** Plasmids, strains and primers used in this study.

Plasmids, strains and primers	Relevant characteristics, sources and sequences	
**Plasmids and strains**	**Relevant characteristics Sources**	
pETDuet_–1_-MCSII*adh*	*adh* (no His⋅Tag), Amp*^r^*	Boshang (Jinan, China)
*E. coli* DH5α	For gene cloning	Weidi (Shanghai, China)
*E. coli* BL21 star (DE3)	For gene expression	Weidi (Shanghai, China)
pET22b-*adh*	*adh* (His⋅Tag), Amp*^r^*	This study
*E. coli* DH5α-pET22b-*adh*	For plasmid cloning	This study
*E. coli* BL21 star (DE3)-pET22b	Empty plasmid pET22b	This study
*E. coli* BL21 star (DE3)-pET22b-*adh*	ADH protein	This study
**Primers**	**Sequences (5′–3′)**	
*adh*-pET22b-*Nde* I-U	GGGAATTCCATATG^*^GCCCAGGCCCTGGTGCTGGAAAAG	
*adh*-pET22b-*Xho* I-D	CCGCTCGAGCAGAACAATCTGCAGTTTAACATC	

*Underlines refer to enzyme restriction sites.

### Media and Cultivation Conditions

The seed culture used in this study was the LB medium containing 10 g/L tryptone, 5 g/L yeast extract, and 10 g/L NaCl. The LB medium supplied with 5 g/L glucose (named LBG medium) was used for the expression of ADH. The cultivation broth of recombinant *E. coli* expressing ADH was inoculated with 1% dose into the LBG medium containing 100 μg/ml ampicillin, and cultivated at 37°C and 200 rpm. After 3 h of cultivation, IPTG was added to the final concentration of 0.2 mM and the cultivation was continued for a further 12 h at 20°C and 100 rpm. The cells of the recombinant *E. coli* expressing ADH were harvested by centrifugation at 4°C and 10,000 × g for 5 min.

### Crude Alcohol Dehydrogenase Preparation, Alcohol Dehydrogenase Purification, and Enzyme Assay

The harvested cells were washed three times by using 20 mM Na_2_HPO_4_-NaH_2_PO_4_ buffer (pH 7.0). The washed cells were collected by centrifugation, resuspended in 20 mM Na_2_HPO_4_-NaH_2_PO_4_ buffer (pH 7.0), and disrupted by sonication at 4°C until the mixture solution became transparent. The supernatant was obtained by centrifugation at 4°C and 10,000 × g for 15 min and was used for crude ADH. The crude ADH was checked by Sodium Dodecyl Sulfate PolyAcrylamide Gel Electrophoresis (SDS–PAGE).

The preparation of crude ADH used for ADH purification is the same as the above except the washing buffer and resuspending buffer were changed to the binding buffer (20 mM NaH_2_PO_4_, 500 mM NaCl, 30 mM imidazole, pH 7.4). HisTrap™ HP (5 mL) column was used for the purification of the recombinant ADH. The column was washed using double-distilled water and equilibrated with a binding buffer. And then, the collected supernatant was loaded onto the column, and the unbound proteins were washed with the binding buffer, and the ADH was then washed with the elution buffer (20 mM NaH_2_PO_4_, 500 mM NaCl, 200 mM imidazole, pH 7.4). Finally, the purified ADH was checked by SDS–PAGE and was concentrated by the ultrafiltration tube with the membrane of the cutoff molecular weight of 10 kDa at 4°C and 3,700 × g. All purification steps of ADH were handled at 4°C.

The 1 ml reaction mixture for ADH assay consisted of each of the following reagents unless otherwise specified: 20 mM Na_2_HPO_4_-NaH_2_PO_4_ buffer (pH 7.0), 2 mM NAD^+^, enzyme solution, and 50 mM allitol, and then incubated at 50°C and 200 rpm shaker for 30 min. One unit of enzyme activity was defined as the amount of D-allulose produced from allitol per minute. The amount of allitol and D-allulose were measured by HPLC using a Carbomix Pb-NP column (7.8 mm × 300 mm, 10 μm, Sepax Technologies) at 78°C and eluted with double-distilled water at a flow rate of 0.5 ml/min.

### Effects of pH, Temperature, and Metal Ions on Recombinant Alcohol Dehydrogenase and Kinetic Modeling

Four buffer systems of sodium acetate–acetic acid (20 mM, pH 5.0–6.0), disodium hydrogen phosphate–sodium dihydrogen phosphate (20 mM, pH 6.0–8.0), tris–HCl (20 mM, pH 8.0–9.0), and glycine–NaOH (20 mM, pH 9.0–11.0) were, respectively, used in determining the optimum pH of the recombinant ADH expressed by *E. coli*.

The optimum temperature for the enzyme activity was measured by assaying the enzyme solution over the temperature range of 30–60°C. The thermal stability of the recombinant ADH was investigated by maintaining the enzyme solution in disodium hydrogen phosphate–sodium dihydrogen phosphate (20 mM, pH 7.0) at various temperatures for 3 h and measuring the residual enzyme activities at 0.5-h intervals.

The residual activity of the enzyme was determined as described in the above method in the “Crude ADH preparation, ADH purification, and enzyme assay.” The enzyme solution was incubated with the metal ions Co^2+^, Zn^2+^, Ni^2+^, Ca^2+^, Mg^2+^, Ba^2+^, Fe^3+^, Mn^2+^, Fe^2+^, and Cu^2+^ at a final concentration of 1 mM. The measured activities were compared with the activity of the enzyme without the metal ion addition (control) under the same conditions.

Kinetic modeling can help to understand the reaction characteristics of this enzyme and predict the reaction results. The reaction rate is normally affected by the substrate concentration, while it is also strongly affected by Co^2+^ for the ADH under investigation. Here, the D-allulose production kinetics under various substrate concentrations of 50, 150, and 250 mM allitol, respectively, with or without the activator of Co^2+^ addition, were investigated.

### D-Allulose Identification

The product was identified by using the HPLC analysis, specific optical rotations, and mass spectrometry. The high performance liquid chromatography (HPLC) analysis method was referred to in “Crude ADH preparation, ADH purification, and enzyme assay.” Specific optical rotations were determined by using the polarimeter (INESA WZZ-3, China). Mass spectrum (BRUKER impactHD, Germany) was performed in the negative ion detection mode with the ESI ion source.

## Results and Discussion

### Cloning, Expression, Purification, and Application of Recombinant *Gluconobacter frateurii* NBRC 3264 Alcohol Dehydrogenase

**FIGURE 2:**
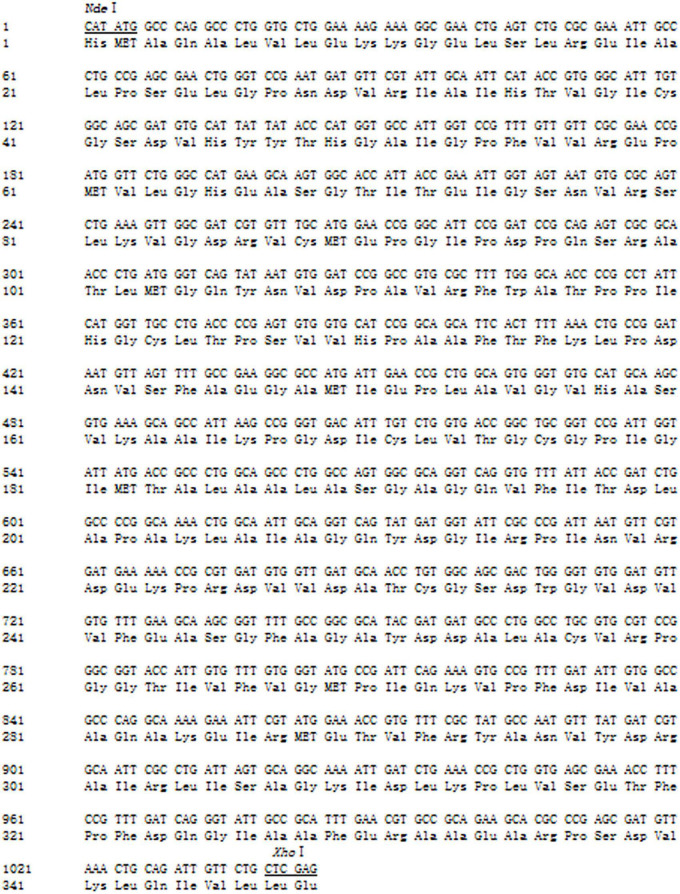
The amino acid sequence and optimized gene sequence of ADH.

**FIGURE 3:**
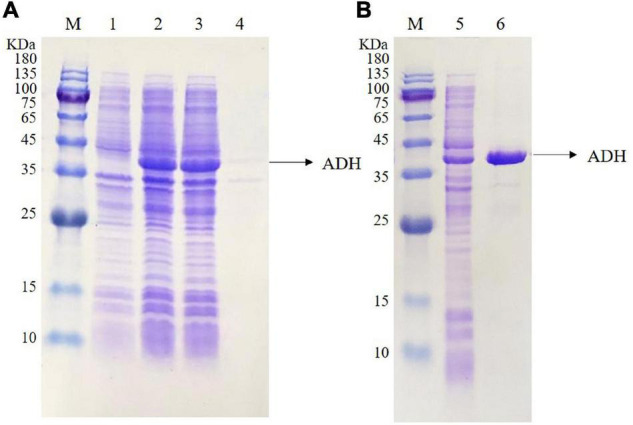
Sodium Dodecyl Sulfate PolyAcrylamide Gel Electrophoresis (SDS-PAGE) analysis of the expressed ADH **(A)** and the purified ADH **(B)**. Lane M, protein marker; lane 1, the total proteins of *E. coli* BL21 star (DE3)-pET22b; lane 2, the total proteins of *E. coli* BL21 star (DE3)-pET22b-*adh*; lane 3, the soluble supernatant of *E. coli* BL21 star (DE3)-pET22b-*adh*; lane 4, the inclusion body of *E. coli* BL21 star (DE3)-pET22b-*adh*; lane 5, ADH crude enzyme solution; lane 6, ADH purified enzyme solution.

The *adh* gene was optimized and synthesized and cloned into pET22b to obtain the recombinant plasmid pET22b-*adh*, which was transformed into *E. coli* BL21 star (DE3). The amino acid sequence (345aa) and the optimized gene sequence of ADH are shown in Figure 2. The recombinant ADH expression was induced by IPTG. The SDS–PAGE analysis showed a strong extra protein band with a molecular mass of ∼36.5 kDa compared with that of the control *E. coli* BL21 star (DE3)-pET22b and confirmed the soluble property of ADH (Figure 3A). The purification of recombinant ADH was carried out by using the HisTrap™ HP (5 mL) column. The result of the ADH purification was analyzed by the SDS–PAGE (Figure 3B), and the purified ADH was concentrated ten times by ultrafiltration.

**FIGURE 4:**
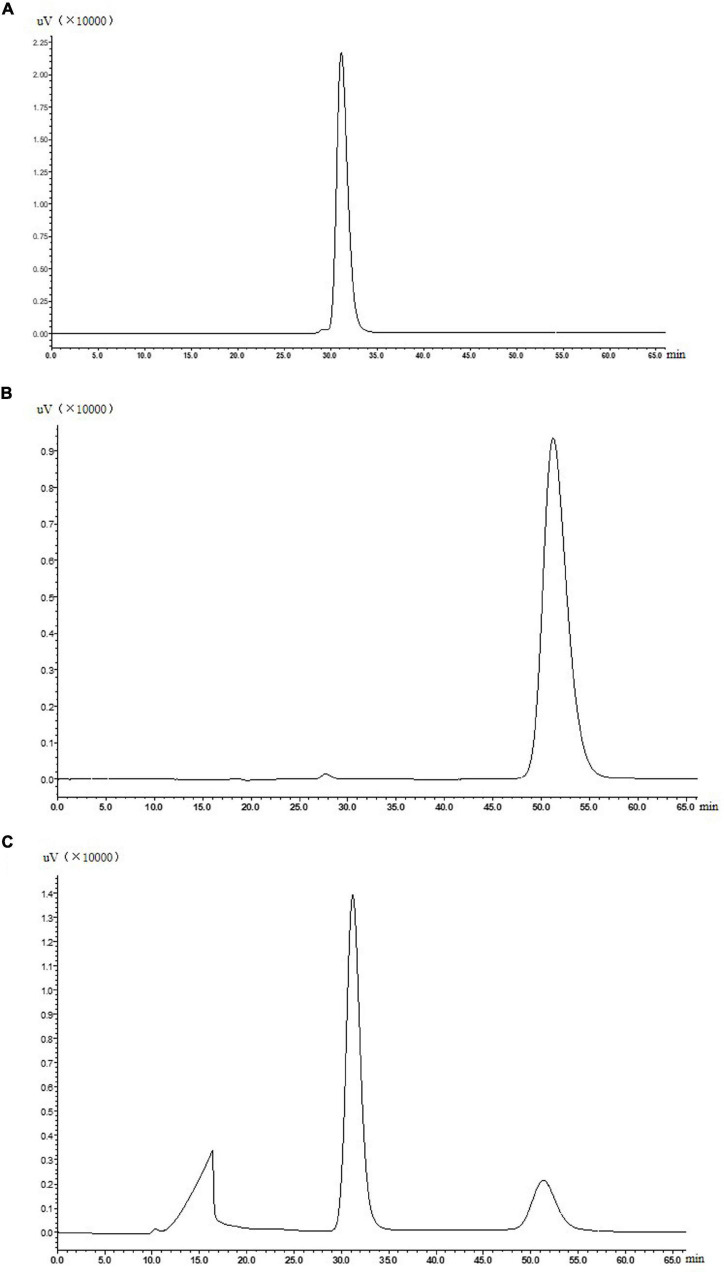
Authentic allitol **(A)**, authentic D-allulose **(B)**, and a sample of reaction solution for the biotransformation of allitol into D-allulose catalyzed by purified ADH **(C)**.

The ADHs catalyze interconversions between alcohols and aldehydes or ketones (Maria-Solano et al., 2017; Zheng et al., 2017; Bartsch et al., 2020). For example, alcohol dehydrogenase from *Pyrococcus furiosus* can catalyze 2, 5-hexanedione to 2, 5-hexanediol (Machielsen et al., 2008). In addition, a sorbitol dehydrogenase (340aa), a homologous enzyme to the alcohol dehydrogenase, which had the same amino acid sequence of ADH from 4 to 343aa, catalyzed the conversion of D-sorbitol to D-fructose in the presence of NAD^+^ (El-Kabbani et al., 2004). The purified and concentrated ADH was inoculated into the reaction solution containing 20 mM Na_2_HPO_4_-NaH_2_PO_4_ buffer (pH 7.0), 2 mM NAD^+^, and 50 mM allitol, and reacted at 50°C shaken at 200 rpm. As shown in Figure 4, the ADH was preliminary confirmed to catalyze allitol into allulose. Next, specific optical rotations of authentic L-allulose, authentic D-allulose, and the purified product were measured. The specific rotation of authentic L-allulose was negative, while the specific rotation of authentic D-allulose and the purified product was positive which agreed with the reports (Gullapalli et al., 2007; Poonperm et al., 2007). Further, the purified product was analyzed by mass spectrometry with a measured mass of 180.1, which was identical to the molar mass of D-allulose. In conclusion, ADH from *G. frateurii* NBRC 3264 can convert allitol into D-allulose, which is the first reported enzyme with this catalytic ability.

### Effect of pH on D-Allulose Biotransformation by Recombinant Alcohol Dehydrogenase

**FIGURE 5:**
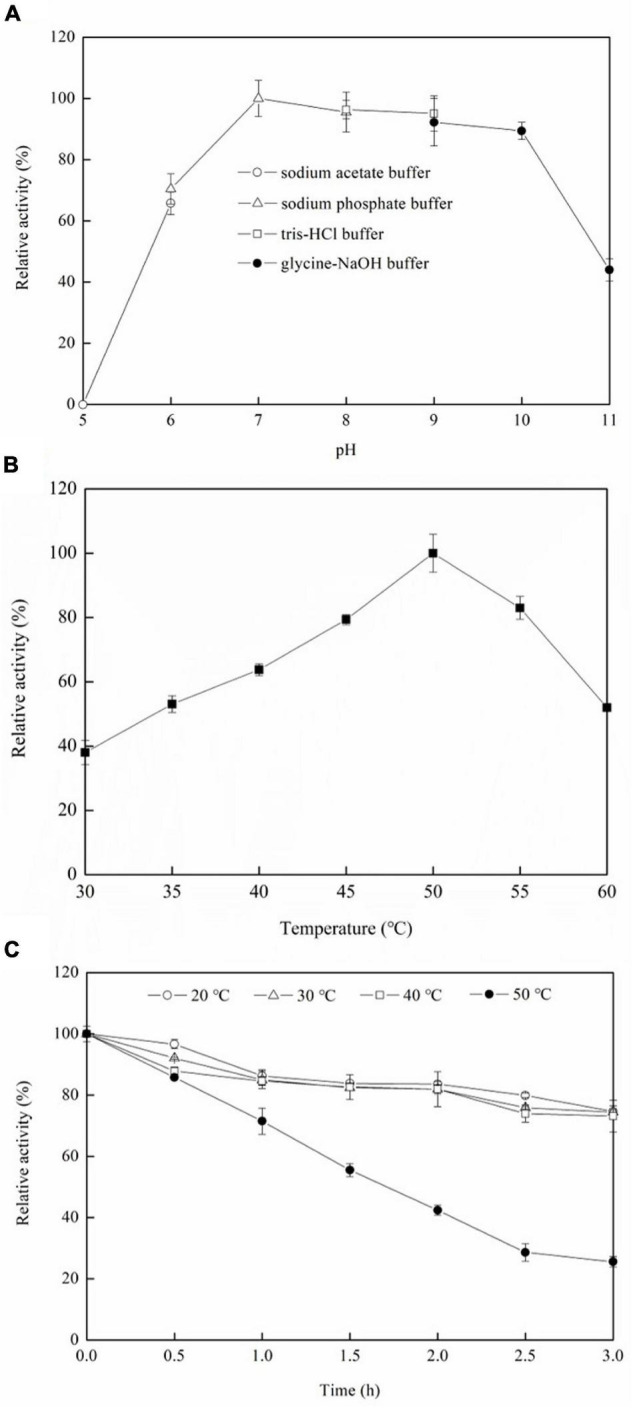
Effects of pH **(A)** and temperature **(B)** on the ADH activities, and the thermal stability of the ADH **(C)**. The conditions for obtaining the highest enzyme activity were set to 100%.

Figure 5A shows that the optimum pH is 7.0, and the relative enzyme activities are above 80% between pH 7.0 and pH 10.0, which indicates that the ADH has a broad pH range. The optimum pH for D-allulose biotransformation from allitol by *Bacillus pallidus* Y25 resting cells was also pH 7.0 (Poonperm et al., 2007). However, the optimum pH for D-allulose biotransformation from allitol by *Enterobacter aerogenes* IK7 was pH 11.0 which was much higher than that of the recombinant ADH (Gullapalli et al., 2007). But, the optimum pH of the enzyme could be different from that of the resting cells in catalyzing the same reaction.

### Effect of Temperature on D-Allulose Biotransformation and Enzyme Stability of the Recombinant Alcohol Dehydrogenase

Figure 5B shows that the optimum temperature is 50°C, and the relative enzyme activities are 63.8, 79.3, 83, and 52% at 40, 45, 55, and 60°C, respectively, compared with that at the optimum temperature. The optimum temperature of *Enterobacter aerogenes* IK7 resting cells for D-allulose biotransformation from allitol was 37°C (Gullapalli et al., 2007), which was lower than that of the recombinant ADH. Nevertheless, the optimum temperature of *Bacillus pallidus* Y25 resting cells for D-allulose biotransformation from allitol was 55°C (Poonperm et al., 2007), which was higher than that of the recombinant ADH.

As seen in Figure 5C, the enzyme has similar thermal stability at 20, 30, and 40°C, and retains 74.7, 74.4, and 73.2% of its initial activity, respectively, after incubation for 3 h at the above temperatures while the enzyme retained 71.5, 42.4, and 25.6% of its initial activity after incubation at 50°C (Figure 5C) for 1, 2, and 3 h, respectively. The results indicated that the ADH had lower thermal stability at a temperature higher than 40°C. Protein engineering is a way to increase the thermal stability of ADH (Magnusson et al., 2019; Zhu et al., 2019b).

### Effect of Metal Ions on D-Allulose Biotransformation by the Recombinant Alcohol Dehydrogenase

**FIGURE 6:**
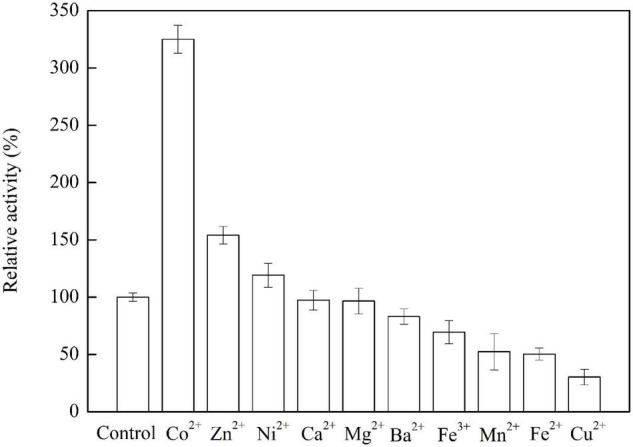
Effects of metal ions on the ADH activity. The conditions for obtaining the highest enzyme activity were set to 100%.

As shown in Figure 6, the addition of Co^2+^, Zn^2+^, or Ni^2+^ increases the enzyme activity by 225, 54.1, and 19.1 %, respectively. It was speculated that Co^2+^ or Ni^2+^ was an activator that can bind to the enzyme and change the enzyme configuration to increase the enzyme activity. It was reported that Zn^2+^ plays an important role in the structure and function of alcohol dehydrogenase and sorbitol dehydrogenase (El-Kabbani et al., 2004). The enzyme activity was slightly decreased by 2.6 and 3.3% when the enzyme was incubated with Ca^2+^ and Mg^2+^, respectively, while the enzyme activity was decreased to 83.1, 69.4, 52.4, 50.3, and 30.3% when the enzyme was incubated with Ba^2+^, Fe^3+^, Mn^2+^, Fe^2+^, and Cu^2+^, respectively. About the activity of NAD-dependent sorbitol dehydrogenase from cold-adapted *Pseudomonas mandelii*, the metal ions of Zn^2+^, Mn^2+^, and Ca^2+^ had slight activation effects while Ni^2+^ had an inhibition effect (DangThu et al., 2021). Ni^2+^, Mn^2+^, Mg^2+^, and Ca^2+^ can increase the ADH activity which was from *Bartonella apis*, while Zn^2+^, Li^+^, and Mo^2+^ decrease the ADH activity (Zhu et al., 2019a). It indicated that the metal-ion-dependence of ADHs derived from different microorganisms was different.

### Effects of Co^2+^ on D-Allulose Biotransformation by the Recombinant Alcohol Dehydrogenase and Kinetic Modeling

**FIGURE 7:**
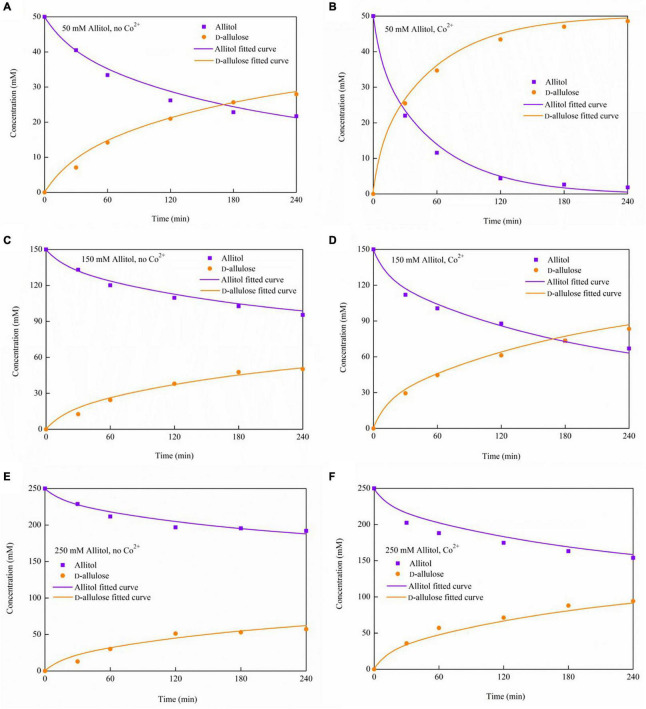
The time courses of allitol and D-allulose concentrations during the biotransformation in the presence or absence of Co^2+^ under various initial allitol concentrations. **(A)** 50 mM allitol, no Co^2+^; **(B)** 50 mM allitol, Co^2+^; **(C)** 150 mM allitol, no Co^2+^; **(D)** 150 mM allitol, Co^2+^; **(E)** 250 mM allitol, no Co^2+^; and **(F)** 250 mM allitol, Co^2+^.

The time courses of D-allulose and allitol concentrations in the presence or absence of Co^2+^ at different allitol concentrations are shown by the dots in Figure 7. The D-allulose conversion yields of 97, 56, and 38%, from the initial allitol concentrations of 50, 150, and 250 mM, respectively, were obtained at 4 h of reaction with 1 mM Co^2+^ added, which was about 1.6-, 1.7-, and 1.7-fold higher, respectively, than that without the Co^2+^ addition.

Then, kinetic modeling was made for D-allulose biotransformation catalyzed by ADH with or without the Co^2+^ addition. Without the Co^2+^ addition, the kinetic equation is shown by Equation (1) and the mass balances are shown by Equations (2) and (3):


(1) $$V=\frac{V_{m\,a\,x}\,S}{\left(k_{s}+S\right)\,\left(1+{\left(P/k_{i}\right)}^{\alpha }\right)}$$

(2) $$\frac{d\,S}{d\,t}=-V$$

(3) $$\frac{d\,P}{d\,t}=V$$

Where, *V*_*max*_, the maximum reaction rate without Co^2+^, mmol/L/h; *k*_*s*_, the substrate affinity constant without Co^2+^, mM; *k*_*i*_, the product inhibition constant, mM; α, constant, (-); *S*, allitol concentration, mM; *P*, D-allulose concentration, mM. With Co^2+^ addition, the kinetic and mass balance equations are as follows:


(4) $$V^{\prime }=\frac{V_{m\,a\,x}\,S}{\left(k_{s}^{\prime }+S\right)\,\left(1+{\left(P/k_{i}\right)}^{\alpha }\right)}$$

(5) $$\frac{d\,S}{d\,t}=-V^{\prime }$$

(6) $$\frac{d\,P}{d\,t}=V^{\prime }$$

**FIGURE 8:**
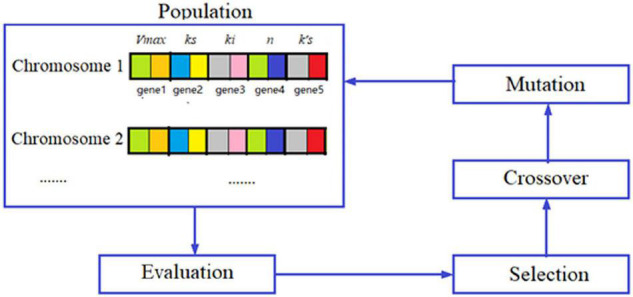
Diagram of the genetic algorithm in parameter value optimization.

Where, *k′_*s*_* is the substrate affinity constant with Co^2+^, mM. The differential equations were solved by using the Runge–Kutta method. The model parameters were obtained by optimization using a genetic algorithm (GA) in minimizing the errors between the model predictions and the measured data, and the optimization diagram is shown in Figure 8. GA is the optimization algorithm that imitates the biological evolutionary processes, which is efficient in solving sophisticated and nonlinear problems. In optimization of the parameter values using GA, one chromosome codes for five genes, and one gene codes for one parameter value as shown in Figure 8. After repeated rounds of biological operations of selection, hybridization (crossover), and mutation until reaching the default termination criteria, the most-fitted chromosome coding for the parameters was obtained to get the optimized parameter values (Figure 8). MatLab 2020b (MathWorks, Inc., United States) running on Windows-compatible personal computer was used in the simulation and model parameter optimization. The optimized model parameter values are shown in Table 2. By using Equations (1)–(6) and the parameter values listed in Table 2 as well as the initial values of allitol concentrations of 50, 150, and 250 mM, respectively, and the initial value of the D-allulose concentration of 0 mM, computer simulation of the biotransformation processes was made and the results are shown by the lines in Figure 7. It showed that the model predictions fitted the experimental data well. It also indicated that the substrate affinity coefficient was much decreased after Co^2+^ addition. Bulut et al. (2020) studied the effect of metal ions on the activity of 10 NAD-dependent formate dehydrogenases and found that there was a clear trend that many metal ions decreased the *Km* values of some FDHs using formate as the substrate, and they estimated that the metal ions could change the protein structure, and the interaction between the substrate or NAD(H) cofactor and the enzyme active site. Therefore, we speculated that the decrease of substrate affinity coefficient after Co^2+^ addition could be the result of the changes of the ADH enzyme structure or the interaction between the substrate of allitol and the active sites of the ADH enzyme. The modeling and simulation results showed that there was product inhibition so that the substrate was hardly completely consumed except in the case at the lowest substrate concentration of 50 mM and at a high enzyme activity with Co^2+^ addition, in which case, the allitol was nearly completely consumed (Figure 7). The modeling and simulation work provided numerical results for the reaction process, which are useful in process analyses and optimizations.

**TABLE 2:** Model parameter values.

Parameter	Value
*V*_*max*_	3.602 mmol/L/h
*ks*	321.809 mM
*ki*	10.740 mM
α	1.326 (−)
*k′s*	21.782 mM

In the conventional method of kinetic modeling, the parameter values of the kinetic equation, like the Michaelis–Menten equation, are first obtained by using double-reciprocal linear plotting. And then, the differential equations are solved for the prediction of the reaction progress. In many cases, the predictions are quite different from the experimental measurements, which indicate that the parameter values obtained this way were not accurate. Therefore, a different method by optimization utilized GA was used in this work, which ensures the accurate prediction of the reaction process. The method using GA was ever successfully applied by us (Lin et al., 2004) and other researchers (Dutta et al., 2005; Yarsky, 2021) in the parameter optimization of the biological models.

## Conclusion

In this study, the gene of NAD(P)-dependent ADH from *G. frateurii* NBRC 3264 was cloned and expressed in *E. coli* BL21 star. The expressed enzyme was purified and was identified for the first time to transform D-allulose from allitol. The effects of pH, temperature, and metal ions on the enzyme activity were determined, and Co^2+^ was found to have a high activation effect on the ADH. A high conversion yield of D-allulose of 97% was obtained at 50 mM allitol with Co^2+^ addition. The kinetics were investigated by modeling and simulation, and product inhibition was found. The enzyme showed enormous potential for application in the high-yield bioconversion of D-allulose and was expected to be applied to the industrial production of D-allulose.

## Data Availability Statement

The datasets presented in this study can be found in online repositories. The names of the repository/repositories and accession number(s) can be found in the article/supplementary material.

## Author Contributions

XW, JianqiL, JianquL, and XS designed the experiments. XW performed the experiments. HL, YN, GL, YR, CL, and CZ gave support to the experiments. XW and JianqiL built the kinetic model, analyzed the data, and wrote the manuscript. All authors read and approved the final manuscript.

## Conflict of Interest

YR was employed by the company Qingdao Longding Biotech Limited Company. The remaining authors declare that the research was conducted in the absence of any commercial or financial relationships that could be construed as a potential conflict of interest.

## Publisher’s Note

All claims expressed in this article are solely those of the authors and do not necessarily represent those of their affiliated organizations, or those of the publisher, the editors and the reviewers. Any product that may be evaluated in this article, or claim that may be made by its manufacturer, is not guaranteed or endorsed by the publisher.

## Footnotes

1
https://www.ncbi.nlm.nih.gov/nuccore/1271388588
